# Nudging Commuters to Increase Public Transport Use: A Field Experiment in Rotterdam

**DOI:** 10.3389/fpsyg.2021.633865

**Published:** 2021-03-11

**Authors:** Samuel Franssens, Ebo Botchway, Willie de Swart, Siegfried Dewitte

**Affiliations:** ^1^Rennes School of Business, Rennes, France; ^2^KU Leuven, Leuven, Belgium; ^3^Rotterdam Elektrische Tram, Rotterdam, Netherlands

**Keywords:** nudge, public transport, social labeling, behavioral science, field experiment

## Abstract

A large-scale field experiment in Rotterdam, Netherlands, tested whether nudging could increase public transport use. During one work week, 4000 commuters on six bus lines, received a free travel card holder. On the three bus lines in the experimental condition, the card holders displayed a social label that branded bus passengers as sustainable travelers because of their bus use. On the three bus lines in the control condition, there was no such message on the card holders. Analysis of the number of rides per hour showed that the intervention led to a change from pre-intervention (619 days) to post-intervention period (176 days) that was estimated to be 1.18 rides per day greater on experimental lines than on control lines. This experiment shows that public transport operators can increase public transport use by incorporating messages that positively label passengers as sustainable travelers in their communication strategies.

## Introduction

Getting people to use public transport more often, instead of cars, will help tackle environmental problems such as air pollution and climate change, road congestion, and traffic accidents. To achieve such behavior change, the transport sector has focused on hard measures such as improvements in physical and technological infrastructure, pollution standards, and pricing mechanisms (Garcia-Sierra et al., 2015). Soft measures, on the other hand, rely on information provision or persuasion to change attitudes, and subsequently, behavior (Bamberg et al., 2011). Research in psychology and behavioral economics has also identified techniques for changing behavior, or so-called nudges, that do not necessarily rely on the intermediate step of changing attitudes.

A nudge refers to “any aspect of the choice architecture that alters people’s behavior in a predictable way without forbidding any options or significantly changing their economic incentives” (Thaler and Sunstein, 2009, p. 6). Nudges help people carry out desirable behaviors by making those behaviors easier and more attractive. They have been shown to be effective tools for achieving behavior change in a variety of domains, including sustainability (Hummel and Maedche, 2019; Trudel, 2019; White et al., 2019). For example, informing people that the social norm among their peers is to use less energy, will lead them to conform to their peers and reduce their energy-use (Schultz et al., 2007; Nolan et al., 2008; Allcott, 2011; Bonan et al., 2020). However, the academic literature contains relatively few examples of highly powered randomized controlled trials that test whether nudges can increase sustainable transportation use (Metcalfe and Dolan, 2012; Lehner et al., 2016; Lieberoth et al., 2018; Gravert and Olsson Collentine, 2019; Kristal and Whillans, 2020).

In this paper, we report a large-scale field experiment that tested whether people can be nudged to use the bus more often. The nudge we used is social labeling. This technique frames a desirable choice as an opportunity to claim a socially valued identity, thereby incentivizing the desirable option with positive self-regard (Kraut, 1973; Allen, 1982; Bryan et al., 2011). Recent field experiments have shown, for example, that promoting sustainable products, such as eco-friendly reusable bags or energy-efficient home appliances, as ‘for green consumers,’ increases their sales (Schwartz et al., 2020). Also, when consumers are primed to perceive their preferences for ecologically superior products as evidence of an environmentally friendly identity, they are subsequently more likely to make sustainable choices, even when they preferred the ecologically superior products simply because they were also functionally superior (Cornelissen et al., 2007). This also works when consumers are primed to perceive their relatively mundane sustainable behaviors, such as avoiding littering, as evidence of their environmental concern (Cornelissen et al., 2008; see also Eby et al., 2019). In our experiment, we encouraged bus passengers to see their bus use as evidence of an environmentally friendly identity, and tested whether this would subsequently motivate them to behave in line with this identity and use the bus more often.

## Method

The field experiment was carried out in co-operation with public transport operator Rotterdam Elektrische Tram (RET). The intervention consisted of giving bus passengers free travel card holders with a message that labeled them as environmentally friendly travelers because of their bus use. Records of bus rides allowed us to measure whether this intervention increased actual bus use, rather than self-reported bus use (Kristal and Whillans, 2020). Participants did not know they were taking part in an experiment and therefore were not adapting their behavior to the fact that they were being observed. This will allow for generalization of the results beyond this particular experiment. In addition, a survey was carried out on experimental and control lines to gather more information about passengers’ travel behavior.

### Procedure

#### Intervention

**FIGURE 1:**
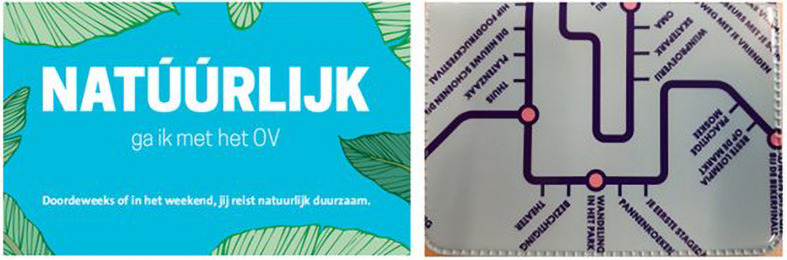
**Left:** The card holder in the experimental condition. **Right:** The card holder in the control condition.

Employees of the public transport operator entered buses on six bus lines and asked passengers whether they wanted a free travel card holder (see Figure 1). On three bus lines, passengers received travel card holders that displayed a message that encouraged them to view the fact that they were taking the bus as evidence of their dedication to traveling sustainably. The original message was in Dutch and was crafted by the marketing department of the public transport operator. It can be translated as ‘Naturally, I use public transport. During the week or during the weekends, it is natural you travel sustainably.’ A pre-test (*N* = 303) confirmed that such a message increases people’s perceptions of the degree to which they take into account the environment in their daily life (compared to people who did not imagine receiving a card holder with this message, on a scale from 1 = ‘I don’t take the environment into account at all’ to 4 = ‘I take the environment into account a lot’: *M*_no_ _message_ = 2.83, *SD* = 0.76 vs. *M*_message_ = 3.01, *SD* = 0.71, *t*(301) = 2.06, *d* = 0.24, *p* = 0.04). On three other bus lines, passengers received a standard card holder distributed by the public transport operator, which displayed no message. One employee per bus line handed out the travel card holders during peak hours for commuters (i.e., between 6 and 10 AM and between 3 and 7 PM), from Monday September 11 until Friday September 15, 2017. During this period, 66253 rides were registered on these lines (30194 on experimental lines and 36059 on control lines; this difference between experimental and control lines is consistent with the pre-intervention difference between these lines). About 4000 travel card holders were distributed during this period, reaching a minimum of 6 percent of passengers. The real percentage is likely to be higher because some passengers will account for more than one ride. A post-intervention survey among passengers (see section “Survey”) estimates it to be 21.5%.

**FIGURE 2:**
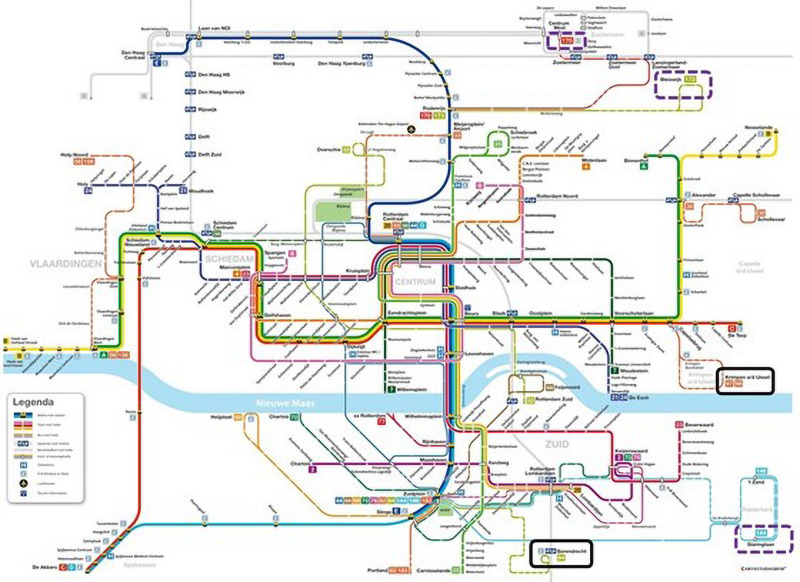
Bus network map of Rotterdam at the time of the experiment. Experimental lines are circled in a solid black line, control lines are circled in a dashed purple line.

The choice of bus lines to include in the experiment was made in consultation with the public transport operator. We initially identified sixteen bus lines that went back and forth between one of Rotterdam’s suburbs and a metro stop where passengers can transfer to Rotterdam city center. We chose three pairs of lines in which the two lines did not overlap geographically and were similar in terms of passengers’ socio-economic background. We then randomly assigned one line of each pair to the experimental treatment (circled in a solid black line: lines 84 in the south; 97 and 98 in the east, these lines travel in opposite directions) and the other to the control treatment (circled in a purple dashed line: lines 144 in the south east; 170 and 173 in the north). Figure 2 shows a map of the bus network in Rotterdam at the time of the experiment. The geographic separation of experimental and control lines was important to reduce the likelihood that bus passengers receiving an experimental card holder would end up riding a control line or vice versa.

#### Survey

To gather more information about the passengers on the bus lines, an extensive survey was conducted during the morning and evening peak hours on the Tuesday and Thursday of the week before and the week after the intervention. Employees of the public transport operator entered buses of the lines included in the experiment and asked randomly chosen passengers to participate in a short survey. One thousand seven hundred and eighty-four passengers agreed to do so (*N*_control,_ _before_ = 465, *N*_control,_ _after_ = 335, *N*_experimental,_ _before_ = 612, *N*_experimental,_ _after_ = 372, but in the following, degrees of freedom will differ across questions because of missing values). Survey questions were the same in the pre-intervention and the post-intervention period, but only in the post-intervention period were passengers asked whether they had received a card holder in the week before (see later). This survey showed that most passengers took the bus five times per week or more (63.2%) or one to four times per week (26.7%). Only small percentages of passengers took the bus one to three times per month (6.6%), less than once per month (1.9%), or hardly ever (1.6%). Passengers on experimental and control lines did not differ in how often they took public transport (on a scale where 4 = ‘1 to 4 times per week’ and 5 = ‘5 or more times per week’: *M*_control_ = 4.45, *SD* = 0.86 vs. *M*_exp_ = 4.50, *SD* = 0.80, *t*(1749) = 1.22, *d* = 0.06, *p* = 0.223), nor in the degree to which they owned cars (overall percentage = 69.1%, χ(1) = 0.00, *p* = 1) or bicycles (overall percentage = 95.4%, χ(1) = 0.00, *p* = 1). Also, passengers did not differ across conditions in the degree to which they took into account the environment in their daily life (all following questions are on a scale from 1 = ‘strongly disagree’ to 7 = ‘strongly agree’: *M*_control_ = 4.55, *SD* = 1.53 vs. *M*_exp_ = 4.62, *SD* = 1.50, *t*(1733) = 0.99, *d* = 0.05, *p* = 0.325), the degree to which the environment was an important reason for them to take public transport (*M*_control_ = 4.07, *SD* = 1.72 vs. *M*_exp_ = 4.09, *SD* = 1.72, *t*(1676) = 0.23, *d* = 0.01, *p* = 0.817), or the degree to which they would like to take public transport more often to go to work or school (*M*_control_ = 4.20, *SD* = 1.90 vs. *M*_exp_ = 4.24, *SD* = 1.97, *t*(1551) = 0.32, *d* = 0.02, *p* = 0.751). Passengers on experimental (vs. control) lines did find it more important that the public transport company actively worked toward a better environment (*M*_control_ = 5.31, *SD* = 1.66 vs. *M*_exp_ = 5.49, *SD* = 1.56, *t*(1736) = 2.24, *d* = 0.11, *p* = 0.025) and they were also happier about the public transport offering in their area (*M*_control_ = 4.87, *SD* = 1.87 vs. *M*_exp_ = 5.16, *SD* = 1.78, *t*(1737) = 3.35, *d* = 0.16, *p* <.001), but these differences can be considered small. After the intervention, 21.5% of passengers reported that they had received a travel card holder in the week before (no difference between experimental and control lines, χ(1) = 0.00, *p* = 1). Passengers who received a travel card holder did not differ significantly from passengers who did not receive a travel card holder on any of the above measures, except that they took public transport more often (*M*_no_ _holder_ = 4.44, *SD* = 0.82 vs. *M*_holder_ = 4.59, *SD* = 0.76, *t*(610) = 1.97, *d* = 0.19, *p* = 0.049).

#### Public Transport Use

The public transport operator provided records of when passengers tapped in (and out) their travel card on the six bus lines. Because tapping in is mandatory on the RET network, regardless of whether a passenger has a long-term pass or buys a one-off ticket, this is a very precise measure of public transport use. Because the data are anonymous, we could not link rides to individual passengers (in other words, ten different rides could result from one passenger taking the bus ten times or from ten different passengers each taking the bus once, or anything in between). The data also did not allow us to distinguish between long-term passes and one-off tickets.

### Analysis

The unit of analysis is the number of rides in 1 hour on a particular bus line. We have data for three experimental lines (84, 97, 98) and three control lines (144, 170, 173), for 791 days, beginning on Friday 2016-01-01 and ending on Saturday 2018-03-10. The intervention started on Monday 2017-09-11 at 6 AM and ended on Friday 2017-09-15 at 7 PM, so we have 619 days of data before the intervention and 176 days of data after the intervention. Most days have 20 h of bus rides. In total, we have *N* = 93083 observations (approximately 6 lines × 791 days × 20 h).

To test whether the change in the number of rides from pre-intervention to post-intervention period is greater in the experimental than in the control condition, we conducted a panel analysis with *condition* (experimental vs. control) and *pre vs. post-intervention period* (post is one day after the intervention or later, pre otherwise) as independent variables. Panel analysis fits a regression model to data sets with a cross-sectional dimension (bus lines) and a longitudinal dimension (time). To obtain a balanced data set, we retain only those hours for which we have a measurement for every line. This results in a data set with 91326 observations (1757 observations or 1.89% were removed). In the statistical model, we include some variables to control for differences between lines and time periods. A random effect of *bus line* (nested within *condition*) will control for differences in the number of rides between bus lines. Fixed effects of *month, weekday*, and *hour* will control for the fact that some months are busier than others, that working days are busier than Saturdays and Sundays, and that certain hours of the day have more rides than others. A main effect of *year* and the interaction between *year* and *month* will allow for changes in the overall level of public transport use across time. An interaction between *weekday* and *hour* will account for the fact that, for example, differences between peak and off-peak hours are larger on working days than during weekends. An interaction between *month* and *weekday* will account for the fact that, for example, differences between working days and weekends are larger in September than in August. Graphical inspection of the data also showed that the difference between experimental and control lines is larger during weekends than during working days. Therefore, an interaction between *line* and *weekday* is included in the model. The residuals of the model with these control variables were normally distributed, but the variance was larger on working days than during weekends. Therefore, we took the logarithm of the number of rides as dependent variable, which appears to solve this problem. Adding the independent variables, we arrive at the following model:

$$\log\,\left(Y_{\mathrm{it}}+1\right)={\mathrm{year}}_{\mathrm{it}}+{\mathrm{month}}_{\mathrm{it}}+{\mathrm{weekday}}_{\mathrm{it}}+{\mathrm{hour}}_{\mathrm{it}}+{\mathrm{year}}_{\mathrm{it}}\times {\mathrm{month}}_{\mathrm{it}}+{\mathrm{month}}_{\mathrm{it}}\times {\mathrm{weekday}}_{\mathrm{it}}+{\mathrm{weekday}}_{\mathrm{it}}\times {\mathrm{hour}}_{\mathrm{it}}+{\mathrm{line}}_{i}+{\mathrm{line}}_{i}\times {\mathrm{weekday}}_{\mathrm{it}}+{\mathrm{condition}}_{i}+{\mathrm{period}}_{\mathrm{it}}+{\mathrm{condition}}_{i}\times {\mathrm{period}}_{\mathrm{it}}+\varepsilon_{i}$$

where *i* refers to bus line and *t* refers to time.

## Results

Panel analysis shows that the interaction between *condition* (0 = control, 1 = treatment) × *period* (0 = pre-intervention, 1 = post-intervention) is significantly positive (*estimate* = 0.057, *t*(91058), = 7.75, *p* < 0.001), indicating that the change in bus use from pre- to post-intervention period was more positive on the experimental than on the control lines. Conversion of the estimate of this interaction term to the original scale gives an effect size of 0.059 extra rides per hour on the experimental (vs. control) lines or 1.18 extra rides on a typical day (0.059 extra rides × 20 h). This constitutes an increase of 0.059%, compared to the average number of rides in the pre-intervention period (100.68 rides per hour or 2013.51 per day). Supplementary Appendix A reports the full results and tests of the assumptions of this analysis.

*Robustness checks*. Supplementary Appendix B shows that the positive interaction effect reported above is robust across models with different specifications, that is, more or fewer interactions between control variables. It also shows that this result is robust across different lengths of the period during which a treatment effect is expected. The period after the intervention can be split into a period immediately after the intervention, during which a treatment effect is expected, and a later period, during which a treatment effect is not expected. The length of the period immediately after the intervention can range from one to 176 days. We carried out the panel analysis reported above for all these possible lengths. Results show that the interaction between condition and period is significantly positive in the period immediately after the intervention, for almost every possible length of the period immediately after the intervention, lowering the likelihood that the effect is due to a random event such as construction works on the control line (unless this happened right after the intervention).

## Discussion

This paper reports a field experiment that showed that giving bus passengers a free travel card holder with a message affirming their identity as sustainable travelers, subsequently led to increased bus use. This intervention was successful even though the subjects in the experiment were not overly motivated by environmental concern in their decisions to take the bus, as evidenced by survey data. Also, the subjects in the experiment were unaware that they were participating in an experiment, so the observed increase in bus use was not driven by subjects’ desire to conform to researchers’ expectations. This increases the generalizability of the results.

Although the obtained increase in bus use may seem small, it is in line with effect sizes from other interventions aimed at encouraging environmentally friendly behavior (Nisa et al., 2019) and it must be interpreted with a few considerations in mind. First, the intervention consisted of only one short message attempting to influence how people see themselves, on a travel card holder that people may or may not have used after receiving it. Also, the card holders were distributed among only an estimated 21.5% of bus passengers during peak hours of one work week (peak hours accounted for 71% of rides during that work week). The effect of such an intervention is bound to be rather small. A simple way to increase the effect size would be to display the message in more or more salient locations, for example, on the travel cards themselves instead of on the holders, or on posters in the buses. This would also help the intervention reach less frequent travelers. Another simple way to increase the effect size would be to distribute more travel card holders. Note, as well, that participants in our experiment were already taking the bus. On the one hand, it is probably easier to convince bus passengers to take the bus more often than it is to convince people to switch to taking the bus for their commute. On the other hand, most bus passengers in our experiment already took the bus regularly, which may have made it harder to convince them to take the bus even more often, placing an upper limit on the effect size. It would be interesting to test whether increases in bus use can be obtained with people who take the bus less often. One factor that may have boosted the effectiveness of the intervention was that passengers on experimental (vs. control) lines attached more importance to the fact that the public transport operator actively worked toward a better environment and were happier about the public transport offering in their neighborhood. This may have made them more receptive to messaging about sustainable traveling coming from the public transport operator. Finally, we did not observe a significant increase or decrease of the effect size across time (see Supplementary Appendix B), but it cannot be ruled out that the effect may become stronger or weaker beyond the time period for which we have data.

Even modest increases in sustainable behaviors can be of significance, especially when it concerns behaviors with a high impact on the environment such as transportation. Furthermore, in this experiment, the cost-benefit ratio of the intervention was quite high, as the public transport operator already distributed free travel card holders on a regular basis (as do many other public transport operators). Information provision is still the most popular strategy among policy-makers for encouraging sustainable behavior. By testing whether insights from behavioral science, integrated into relatively inexpensive marketing actions, can encourage people to use public transport more often, this study demonstrates the value of cooperation between public transport operators and behavioral scientists. Although nudges alone will not achieve the increases in public transport use that are necessary to achieve large reductions in carbon emissions, they will often produce better results than information provision. Recent research has also proposed that nudging may also improve the effectiveness of policy tools such as financial incentives or legislation (Nisa et al., 2019).

This study has some limitations. First, after the intervention, the survey only asked passengers whether they had received a card holder in the week before, not which card holder they had received. Doing so would have allowed us to check whether passengers who had received a card holder were aware of the message on the card holder, which could have functioned as a manipulation check. Second, we tested the effectiveness of the social labeling intervention against a control condition that received card holders with no message. We therefore cannot be sure whether the intervention will also increase bus use compared to a control condition that receives card holders with a positive message, similar to the one we used, but that does not rely on social labeling. A third limitation is that we could not link individual bus passengers with social labels (treatments) and rides (dependent variable), because rides are recorded anonymously. Because of that, there was little point in assigning individuals to treatments and therefore we had to rely on assigning bus lines to treatments. Finally, even though we assigned comparable lines to the treatments and included a number of variables in the model to control for existing patterns of bus use (see Supplementary Material), we cannot rule out that the effect of the intervention was caused by external events not captured by the model. Future research that is able to link passengers with treatments and rides will allow for a more precise test of the treatment effect. When combined with additional survey data, such studies would also make it possible to explore whether increases in bus use are due to (self-reported) decreases in car use and whether the effect of the intervention may spillover to other sustainable actions such as taking the bicycle more often (Fanghella et al., 2019). Such studies will also allow for theoretical advancement on social labeling, such as testing whether the effect of the intervention is mediated by changes in self-perceived identity and whether the intervention works better for people with higher or lower levels of environmental concern.

## Data Availability Statement

The raw data supporting the conclusions of this article will be made available by the authors, without undue reservation.

## Ethics Statement

The studies involving human participants were reviewed and approved by the privacy team Rotterdam Elektrische Tram, hereby represented by the legal professional member (M.G.M. Land) has evaluated the Ciptec Paper “Nudging commuters to increase public transport use: a field experiment in Rotterdam” by authors from Rennes School of Business, KU Leuven and Rotterdam Elektrische Tram. The manuscript is based on a large field experiment in which bus passengers were involved. The method, procedure, data and analysis are set up and performed in an anonymous way. No personal data or confidential information are involved and neither ethical topics are addressed. This leads to the conclusion that the experiment and manuscript succeeds the test of ethic approval. The privacy team, represented by Mrs. Land, herewith gives approval to any use and publication of the manuscript. Written informed consent from the participants’ legal guardian/next of kin was not required to participate in this study in accordance with the national legislation and the institutional requirements.

## Author Contributions

SF: conceptualization, methodology, formal analysis, writing – original draft, writing – review and editing, visualization, and data curation. EB: formal analysis, writing – review and editing. WS: methodology, investigation, writing – review and editing, and funding acquisition. SD: conceptualization, methodology, writing – review and editing, and funding acquisition. All authors contributed to the article and approved the submitted version.

## Conflict of Interest

The authors declare that the research was conducted in the absence of any commercial or financial relationships that could be construed as a potential conflict of interest.
