# Supplementary material for: Nudging Commuters to Increase Public Transport Use: A Field Experiment in Rotterdam
Source: Front Psychol. 2021 Mar 11;12:633865. doi: 10.3389/fpsyg.2021.633865 (PMC7990888; doi:10.3389/fpsyg.2021.633865)

Graph 1: the average, per day, of log(amount of rides per hour +1) across time


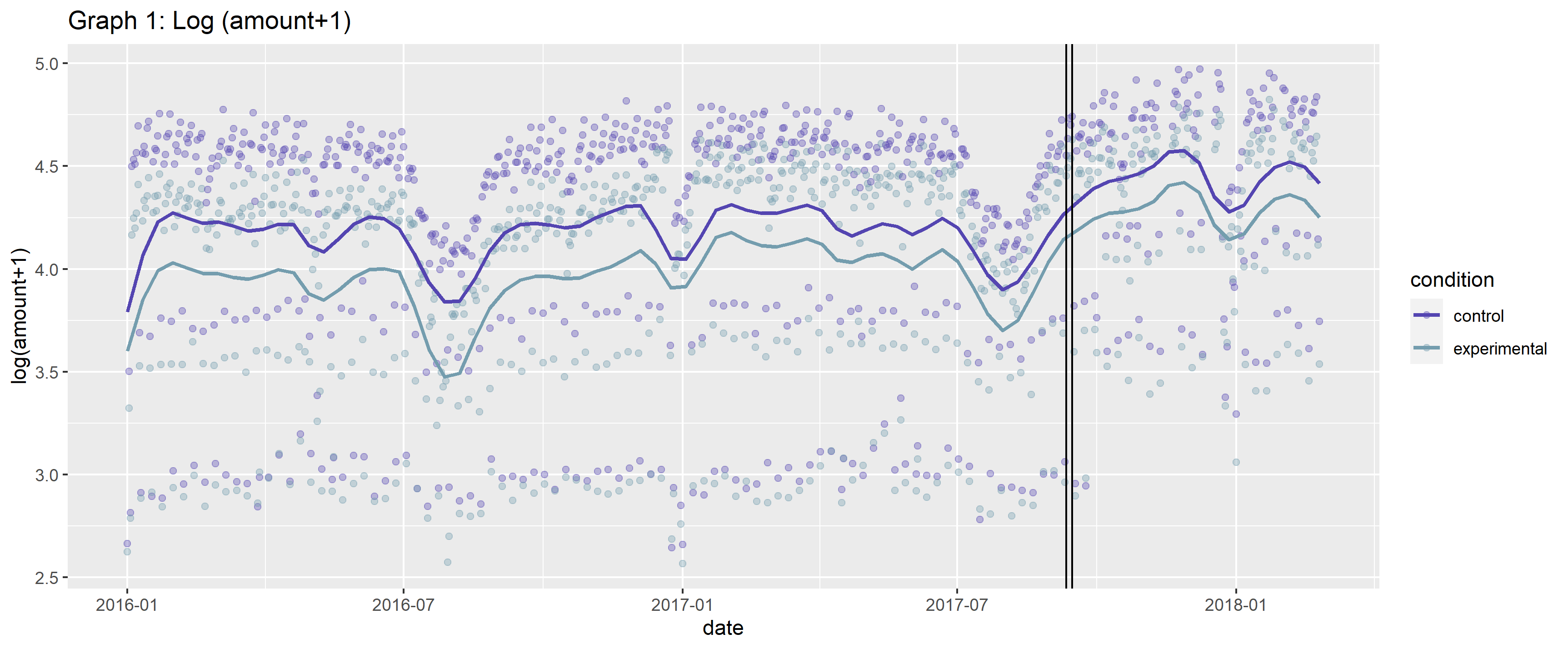


Graph 2: the average, per day, of log(amount of rides per hour +1) across time with the effect of condition removed


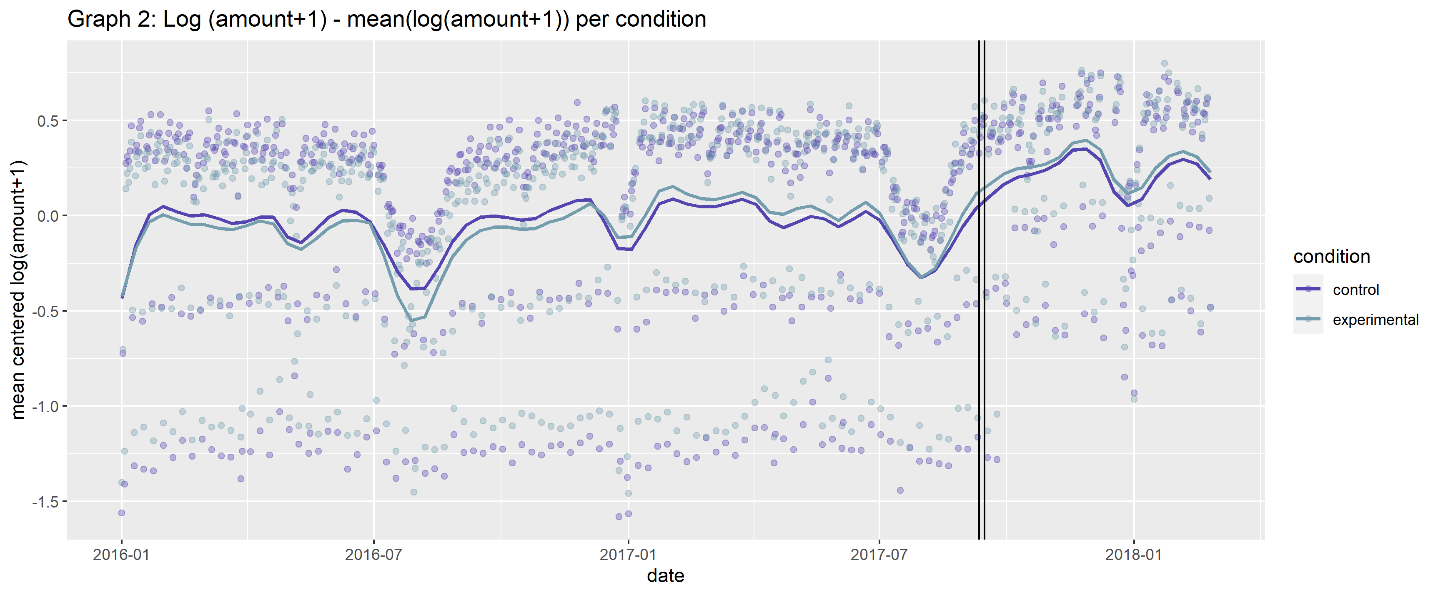


Graph 3a: the average, per day, of the residuals of the model with the control variables, across time


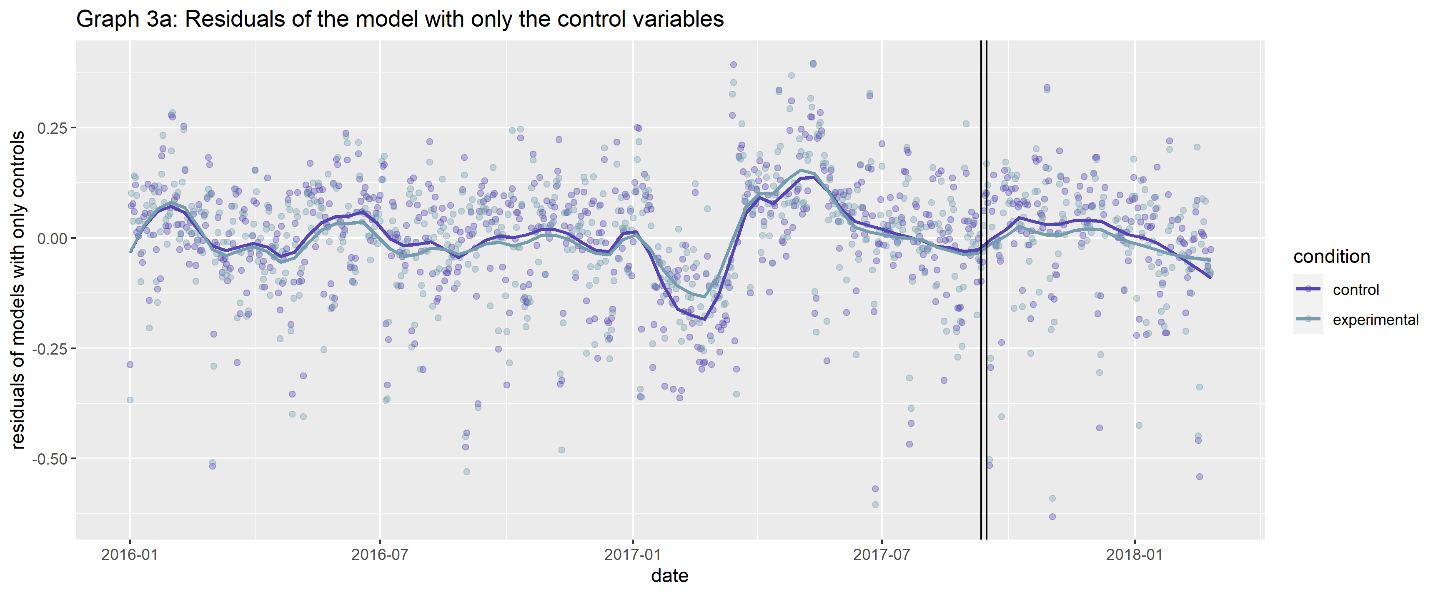


Graph 3b: per hour, the residuals of the model with the control variables, across time


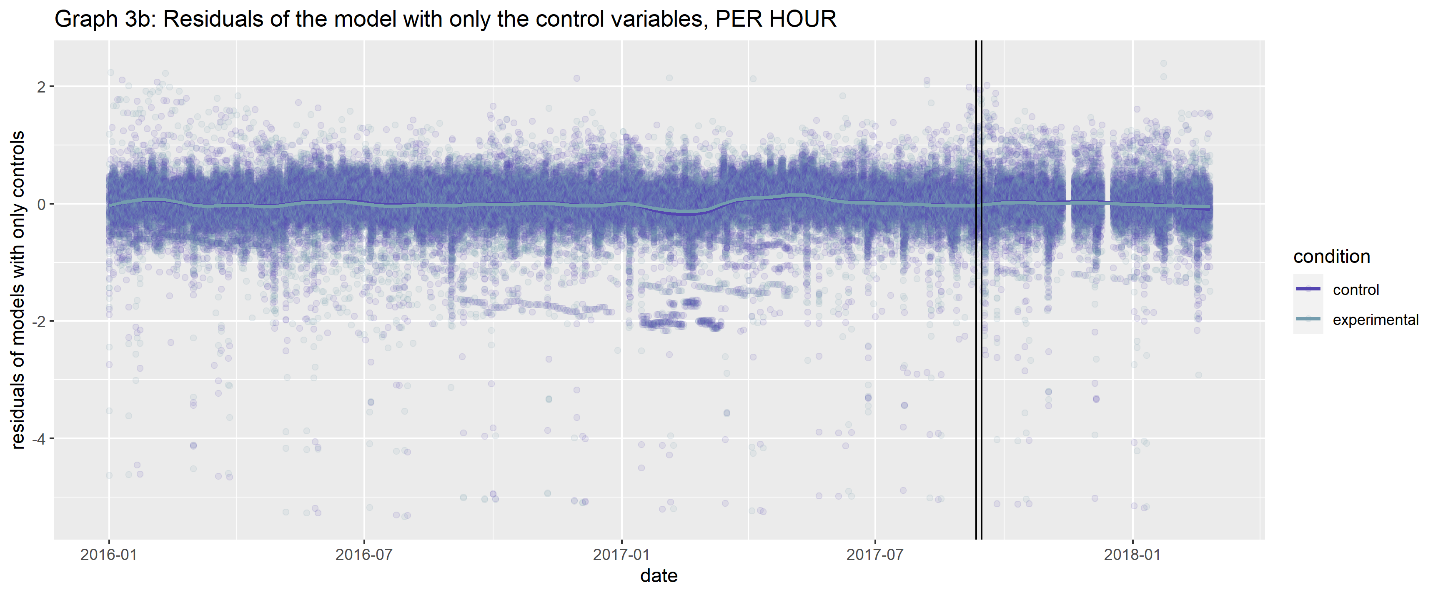

Supplement: Supplementary file 1 [file Data_Sheet_1.docx]
