# Supplementary material for: Nudging Commuters to Increase Public Transport Use: A Field Experiment in Rotterdam
Source: Front Psychol. 2021 Mar 11;12:633865. doi: 10.3389/fpsyg.2021.633865 (PMC7990888; doi:10.3389/fpsyg.2021.633865)
Supplement: Supplementary file 2 [file Data_Sheet_2.docx]

# Appendix A: Bus use analysis: Assumptions and full results of the panel analysis

Any model testing the *condition* (experimental vs. control) $\times$ *period* (pre vs post-intervention) interaction should include some variables to control for existing patterns of bus use. Our data set contains five control variables (*year*, *month*, *weekday*, *hour*, and *line*). In the main text, we identified

$log(Y_{it}+1)=year_{it}+month_{it}+weekday_{it}+hour_{it}+year_{it}\times month_{it}+month_{it}\times weekday_{it}+weekday_{it}\times hour_{it}+line_{i}+line_{i}\times weekday_{it}+\varepsilon_{i}$ (Model 1)

where *i* refers to bus line and *t* refers to time, as the model to control for existing bus use patterns that made most sense to us from a theoretical point of view. We carried out a unit-root test for panel data (Choi, 2001) in R (R Core Team, 2020) with package aTSA (Qiu, 2015) on the residuals of Model 1. This strongly rejects the null hypothesis of unit root, indicating that the set of series is stationary (tested at every lag from 1 to 42, which is the maximum number of hours of bus rides in a two-day period, and testing with no exogenous variables, individual intercepts, and individual intercepts and trends in the augmented Dickey-Fuller regressions). We can therefore carry out a ‘within-between’ panel analysis on the residuals of Model 1. This panel analysis was carried out in R with package panelr (Long, 2020). The resulting test of the interaction between *condition* (0 = control, 1 = treatment) $\times$ *period* (0 = pre-intervention, 1 = post-intervention) is reported in the main text. Below is the full table of regression coefficients:

| term | estimate | standard error | t value | df | p value |
| --- | --- | --- | --- | --- | --- |
| (Intercept) | 4.6373 | 0.1498 | 30.958 | 0 | 0.9997 |
| year2017 | 0.1128 | 0.0047 | 23.851 | 91058 | 0.0000 |
| year2018 | 0.1534 | 0.0172 | 8.938 | 91058 | 0.0000 |
| month2 | 0.0546 | 0.0068 | 8.066 | 91058 | 0.0000 |
| month3 | 0.0306 | 0.0071 | 4.314 | 91058 | 0.0000 |
| month4 | 0.0139 | 0.0071 | 1.953 | 91058 | 0.0508 |
| month5 | -0.0354 | 0.0071 | -4.997 | 91058 | 0.0000 |
| month6 | 0.0199 | 0.0071 | 2.786 | 91058 | 0.0053 |
| month7 | -0.2288 | 0.0071 | -32.285 | 91058 | 0.0000 |
| month8 | -0.2382 | 0.0071 | -33.604 | 91058 | 0.0000 |
| month9 | 0.0344 | 0.0079 | 4.372 | 91058 | 0.0000 |
| month10 | 0.0582 | 0.0097 | 6.026 | 91058 | 0.0000 |
| month11 | 0.1262 | 0.0099 | 12.786 | 91058 | 0.0000 |
| month12 | 0.0152 | 0.0098 | 1.549 | 91058 | 0.1214 |
| weekdaySaturday | -0.7411 | 0.0129 | -57.257 | 91058 | 0.0000 |
| weekdaySunday | -1.4355 | 0.0130 | -110.203 | 91058 | 0.0000 |
| weekdayMonday | -0.2575 | 0.0130 | -19.829 | 91058 | 0.0000 |
| weekdayTuesday | -0.1167 | 0.0130 | -9.006 | 91058 | 0.0000 |
| weekdayWednesday | -0.1159 | 0.0130 | -8.947 | 91058 | 0.0000 |
| weekdayThursday | -0.1117 | 0.0129 | -8.635 | 91058 | 0.0000 |
| hour6 | 1.8184 | 0.0092 | 197.108 | 91058 | 0.0000 |
| hour7 | 2.9194 | 0.0092 | 318.511 | 91058 | 0.0000 |
| hour8 | 3.2110 | 0.0092 | 350.424 | 91058 | 0.0000 |
| hour9 | 2.9539 | 0.0091 | 324.894 | 91058 | 0.0000 |
| hour10 | 2.9093 | 0.0091 | 319.985 | 91058 | 0.0000 |
| hour11 | 3.0594 | 0.0091 | 336.494 | 91058 | 0.0000 |
| hour12 | 3.2353 | 0.0091 | 355.845 | 91058 | 0.0000 |
| hour13 | 3.2887 | 0.0091 | 361.831 | 91058 | 0.0000 |
| hour14 | 3.3380 | 0.0091 | 367.135 | 91058 | 0.0000 |
| hour15 | 3.4934 | 0.0091 | 384.121 | 91058 | 0.0000 |
| hour16 | 3.6143 | 0.0091 | 397.420 | 91058 | 0.0000 |
| hour17 | 3.6342 | 0.0091 | 399.596 | 91058 | 0.0000 |
| hour18 | 3.0736 | 0.0091 | 338.061 | 91058 | 0.0000 |
| hour19 | 2.5162 | 0.0091 | 276.663 | 91058 | 0.0000 |
| hour20 | 2.3112 | 0.0091 | 254.059 | 91058 | 0.0000 |
| hour21 | 2.2422 | 0.0091 | 246.468 | 91058 | 0.0000 |
| hour22 | 2.0662 | 0.0091 | 227.125 | 91058 | 0.0000 |
| hour23 | 1.7345 | 0.0091 | 190.664 | 91058 | 0.0000 |
| hour24 | 0.9075 | 0.0091 | 99.446 | 91058 | 0.0000 |
| prepostpost | -0.0263 | 0.0148 | -1.776 | 91058 | 0.0758 |
| linectrl170 | -0.5141 | 0.2118 | -2.427 | 0 | 0.9998 |
| linectrl173 | -0.7233 | 0.2118 | -3.414 | 0 | 0.9997 |
| lineexp84 | -0.4994 | 0.2118 | -2.357 | 0 | 0.9998 |
| lineexp97 | -0.7179 | 0.2118 | -3.389 | 0 | 0.9997 |
| lineexp98 | -0.6209 | 0.2118 | -2.931 | 0 | 0.9998 |
| weekdaySaturday:linectrl170 | -0.2743 | 0.0183 | -14.997 | 91058 | 0.0000 |
| weekdaySaturday:linectrl173 | -0.0646 | 0.0183 | -3.530 | 91058 | 0.0004 |
| weekdaySaturday:lineexp84 | -0.0268 | 0.0183 | -1.466 | 91058 | 0.1425 |
| weekdaySaturday:lineexp97 | 0.0592 | 0.0183 | 3.235 | 91058 | 0.0012 |
| weekdaySaturday:lineexp98 | -0.1788 | 0.0183 | -9.775 | 91058 | 0.0000 |
| weekdaySunday:linectrl170 | -0.2743 | 0.0184 | -14.904 | 91058 | 0.0000 |
| weekdaySunday:linectrl173 | -0.2138 | 0.0184 | -11.615 | 91058 | 0.0000 |
| weekdaySunday:lineexp84 | 0.1720 | 0.0184 | 9.345 | 91058 | 0.0000 |
| weekdaySunday:lineexp97 | -0.0802 | 0.0184 | -4.355 | 91058 | 0.0000 |
| weekdaySunday:lineexp98 | -0.0891 | 0.0184 | -4.840 | 91058 | 0.0000 |
| weekdayMonday:linectrl170 | 0.0409 | 0.0184 | 2.231 | 91058 | 0.0257 |
| weekdayMonday:linectrl173 | 0.0728 | 0.0184 | 3.967 | 91058 | 0.0001 |
| weekdayMonday:lineexp84 | 0.0334 | 0.0184 | 1.821 | 91058 | 0.0686 |
| weekdayMonday:lineexp97 | 0.0336 | 0.0184 | 1.830 | 91058 | 0.0673 |
| weekdayMonday:lineexp98 | 0.0370 | 0.0184 | 2.014 | 91058 | 0.0440 |
| weekdayTuesday:linectrl170 | 0.0542 | 0.0183 | 2.959 | 91058 | 0.0031 |
| weekdayTuesday:linectrl173 | 0.0691 | 0.0183 | 3.772 | 91058 | 0.0002 |
| weekdayTuesday:lineexp84 | 0.0154 | 0.0183 | 0.839 | 91058 | 0.4012 |
| weekdayTuesday:lineexp97 | 0.0740 | 0.0183 | 4.040 | 91058 | 0.0001 |
| weekdayTuesday:lineexp98 | 0.0744 | 0.0183 | 4.061 | 91058 | 0.0000 |
| weekdayWednesday:linectrl170 | 0.0395 | 0.0183 | 2.159 | 91058 | 0.0309 |
| weekdayWednesday:linectrl173 | 0.0587 | 0.0183 | 3.207 | 91058 | 0.0013 |
| weekdayWednesday:lineexp84 | -0.0270 | 0.0183 | -1.477 | 91058 | 0.1397 |
| weekdayWednesday:lineexp97 | 0.0340 | 0.0183 | 1.857 | 91058 | 0.0633 |
| weekdayWednesday:lineexp98 | 0.0336 | 0.0183 | 1.837 | 91058 | 0.0662 |
| weekdayThursday:linectrl170 | 0.0717 | 0.0183 | 3.924 | 91058 | 0.0001 |
| weekdayThursday:linectrl173 | 0.0937 | 0.0183 | 5.125 | 91058 | 0.0000 |
| weekdayThursday:lineexp84 | 0.0265 | 0.0183 | 1.450 | 91058 | 0.1470 |
| weekdayThursday:lineexp97 | 0.0566 | 0.0183 | 3.095 | 91058 | 0.0020 |
| weekdayThursday:lineexp98 | 0.0656 | 0.0183 | 3.590 | 91058 | 0.0003 |
| conditionexperimental:prepostpost | 0.0573 | 0.0074 | 7.752 | 91058 | 0.0000 |
| year2017:month2 | -0.0013 | 0.0144 | -0.088 | 91058 | 0.9299 |
| year2017:month3 | 0.0311 | 0.0141 | 2.207 | 91058 | 0.0273 |
| year2017:month4 | -0.1185 | 0.0142 | -8.335 | 91058 | 0.0000 |
| year2017:month5 | 0.0383 | 0.0141 | 2.720 | 91058 | 0.0065 |
| year2017:month6 | -0.0758 | 0.0142 | -5.333 | 91058 | 0.0000 |
| year2017:month7 | 0.0229 | 0.0141 | 1.624 | 91058 | 0.1044 |
| year2017:month8 | 0.0024 | 0.0141 | 0.172 | 91058 | 0.8632 |
| year2017:month9 | 0.0417 | 0.0160 | 2.610 | 91058 | 0.0091 |
| year2017:month10 | 0.0513 | 0.0202 | 2.537 | 91058 | 0.0112 |
| year2017:month11 | 0.0660 | 0.0207 | 3.196 | 91058 | 0.0014 |
| year2017:month12 | 0.0211 | 0.0206 | 1.024 | 91058 | 0.3057 |
| year2018:month2 | 0.0522 | 0.0151 | 3.461 | 91058 | 0.0005 |
| month2:weekdaySaturday | -0.1139 | 0.0224 | -5.093 | 91058 | 0.0000 |
| month2:weekdaySunday | -0.1003 | 0.0224 | -4.478 | 91058 | 0.0000 |
| month2:weekdayMonday | -0.0616 | 0.0223 | -2.768 | 91058 | 0.0056 |
| month2:weekdayTuesday | -0.0993 | 0.0225 | -4.416 | 91058 | 0.0000 |
| month2:weekdayWednesday | -0.1199 | 0.0227 | -5.285 | 91058 | 0.0000 |
| month2:weekdayThursday | -0.1007 | 0.0226 | -4.462 | 91058 | 0.0000 |
| month3:weekdaySaturday | -0.0566 | 0.0246 | -2.306 | 91058 | 0.0211 |
| month3:weekdaySunday | -0.0585 | 0.0245 | -2.391 | 91058 | 0.0168 |
| month3:weekdayMonday | -0.1746 | 0.0245 | -7.116 | 91058 | 0.0000 |
| month3:weekdayTuesday | -0.0849 | 0.0241 | -3.526 | 91058 | 0.0004 |
| month3:weekdayWednesday | -0.1035 | 0.0239 | -4.331 | 91058 | 0.0000 |
| month3:weekdayThursday | -0.0871 | 0.0240 | -3.624 | 91058 | 0.0003 |
| month4:weekdaySaturday | -0.0096 | 0.0238 | -0.402 | 91058 | 0.6877 |
| month4:weekdaySunday | 0.0893 | 0.0240 | 3.715 | 91058 | 0.0002 |
| month4:weekdayMonday | -0.1574 | 0.0245 | -6.417 | 91058 | 0.0000 |
| month4:weekdayTuesday | -0.0257 | 0.0245 | -1.048 | 91058 | 0.2948 |
| month4:weekdayWednesday | -0.1346 | 0.0247 | -5.451 | 91058 | 0.0000 |
| month4:weekdayThursday | -0.1346 | 0.0248 | -5.426 | 91058 | 0.0000 |
| month5:weekdaySaturday | 0.0211 | 0.0250 | 0.843 | 91058 | 0.3993 |
| month5:weekdaySunday | 0.1491 | 0.0245 | 6.098 | 91058 | 0.0000 |
| month5:weekdayMonday | -0.0630 | 0.0242 | -2.606 | 91058 | 0.0092 |
| month5:weekdayTuesday | -0.0053 | 0.0241 | -0.221 | 91058 | 0.8252 |
| month5:weekdayWednesday | -0.0222 | 0.0247 | -0.900 | 91058 | 0.3682 |
| month5:weekdayThursday | -0.3093 | 0.0252 | -12.258 | 91058 | 0.0000 |
| month6:weekdaySaturday | -0.0388 | 0.0246 | -1.582 | 91058 | 0.1136 |
| month6:weekdaySunday | -0.0131 | 0.0245 | -0.534 | 91058 | 0.5934 |
| month6:weekdayMonday | -0.2085 | 0.0245 | -8.501 | 91058 | 0.0000 |
| month6:weekdayTuesday | -0.1149 | 0.0245 | -4.689 | 91058 | 0.0000 |
| month6:weekdayWednesday | -0.1227 | 0.0243 | -5.054 | 91058 | 0.0000 |
| month6:weekdayThursday | -0.1156 | 0.0240 | -4.811 | 91058 | 0.0000 |
| month7:weekdaySaturday | 0.1059 | 0.0238 | 4.457 | 91058 | 0.0000 |
| month7:weekdaySunday | 0.2772 | 0.0237 | 11.717 | 91058 | 0.0000 |
| month7:weekdayMonday | -0.0250 | 0.0241 | -1.039 | 91058 | 0.2989 |
| month7:weekdayTuesday | -0.0700 | 0.0245 | -2.858 | 91058 | 0.0043 |
| month7:weekdayWednesday | -0.0961 | 0.0247 | -3.891 | 91058 | 0.0001 |
| month7:weekdayThursday | -0.1009 | 0.0248 | -4.068 | 91058 | 0.0000 |
| month8:weekdaySaturday | 0.1610 | 0.0250 | 6.445 | 91058 | 0.0000 |
| month8:weekdaySunday | 0.2935 | 0.0249 | 11.799 | 91058 | 0.0000 |
| month8:weekdayMonday | 0.0096 | 0.0245 | 0.391 | 91058 | 0.6958 |
| month8:weekdayTuesday | -0.0375 | 0.0241 | -1.551 | 91058 | 0.1209 |
| month8:weekdayWednesday | -0.0553 | 0.0243 | -2.272 | 91058 | 0.0231 |
| month8:weekdayThursday | -0.0755 | 0.0248 | -3.045 | 91058 | 0.0023 |
| month9:weekdaySaturday | -0.0634 | 0.0238 | -2.666 | 91058 | 0.0077 |
| month9:weekdaySunday | -0.0338 | 0.0241 | -1.404 | 91058 | 0.1604 |
| month9:weekdayMonday | -0.0641 | 0.0242 | -2.653 | 91058 | 0.0080 |
| month9:weekdayTuesday | -0.1229 | 0.0241 | -5.090 | 91058 | 0.0000 |
| month9:weekdayWednesday | -0.1251 | 0.0243 | -5.142 | 91058 | 0.0000 |
| month9:weekdayThursday | -0.1182 | 0.0240 | -4.918 | 91058 | 0.0000 |
| month10:weekdaySaturday | -0.0694 | 0.0247 | -2.807 | 91058 | 0.0050 |
| month10:weekdaySunday | -0.0463 | 0.0245 | -1.892 | 91058 | 0.0585 |
| month10:weekdayMonday | -0.1083 | 0.0245 | -4.427 | 91058 | 0.0000 |
| month10:weekdayTuesday | -0.1291 | 0.0245 | -5.267 | 91058 | 0.0000 |
| month10:weekdayWednesday | -0.1516 | 0.0251 | -6.037 | 91058 | 0.0000 |
| month10:weekdayThursday | -0.1283 | 0.0252 | -5.086 | 91058 | 0.0000 |
| month11:weekdaySaturday | -0.1286 | 0.0262 | -4.901 | 91058 | 0.0000 |
| month11:weekdaySunday | -0.1242 | 0.0263 | -4.715 | 91058 | 0.0000 |
| month11:weekdayMonday | -0.0483 | 0.0261 | -1.853 | 91058 | 0.0639 |
| month11:weekdayTuesday | -0.1410 | 0.0255 | -5.527 | 91058 | 0.0000 |
| month11:weekdayWednesday | -0.1593 | 0.0252 | -6.308 | 91058 | 0.0000 |
| month11:weekdayThursday | -0.1321 | 0.0258 | -5.123 | 91058 | 0.0000 |
| month12:weekdaySaturday | -0.1686 | 0.0243 | -6.947 | 91058 | 0.0000 |
| month12:weekdaySunday | -0.0586 | 0.0251 | -2.333 | 91058 | 0.0196 |
| month12:weekdayMonday | -0.3268 | 0.0252 | -12.962 | 91058 | 0.0000 |
| month12:weekdayTuesday | -0.2098 | 0.0252 | -8.331 | 91058 | 0.0000 |
| month12:weekdayWednesday | -0.1537 | 0.0253 | -6.083 | 91058 | 0.0000 |
| month12:weekdayThursday | -0.1001 | 0.0248 | -4.032 | 91058 | 0.0001 |
| weekdaySaturday:hour6 | -0.7910 | 0.0349 | -22.651 | 91058 | 0.0000 |
| weekdaySaturday:hour7 | -0.1451 | 0.0341 | -4.253 | 91058 | 0.0000 |
| weekdaySaturday:hour8 | 0.3228 | 0.0341 | 9.462 | 91058 | 0.0000 |
| weekdaySaturday:hour9 | 1.1075 | 0.0341 | 32.468 | 91058 | 0.0000 |
| weekdaySaturday:hour10 | 1.4217 | 0.0341 | 41.677 | 91058 | 0.0000 |
| weekdaySaturday:hour11 | 1.6180 | 0.0341 | 47.431 | 91058 | 0.0000 |
| weekdaySaturday:hour12 | 1.5551 | 0.0341 | 45.588 | 91058 | 0.0000 |
| weekdaySaturday:hour13 | 1.6355 | 0.0341 | 47.944 | 91058 | 0.0000 |
| weekdaySaturday:hour14 | 1.5135 | 0.0341 | 44.369 | 91058 | 0.0000 |
| weekdaySaturday:hour15 | 1.2796 | 0.0341 | 37.511 | 91058 | 0.0000 |
| weekdaySaturday:hour16 | 1.1982 | 0.0341 | 35.124 | 91058 | 0.0000 |
| weekdaySaturday:hour17 | 1.1359 | 0.0341 | 33.299 | 91058 | 0.0000 |
| weekdaySaturday:hour18 | 1.3224 | 0.0341 | 38.767 | 91058 | 0.0000 |
| weekdaySaturday:hour19 | 1.5990 | 0.0341 | 46.876 | 91058 | 0.0000 |
| weekdaySaturday:hour20 | 1.6539 | 0.0341 | 48.484 | 91058 | 0.0000 |
| weekdaySaturday:hour21 | 1.5273 | 0.0341 | 44.773 | 91058 | 0.0000 |
| weekdaySaturday:hour22 | 1.7623 | 0.0341 | 51.661 | 91058 | 0.0000 |
| weekdaySaturday:hour23 | 1.9717 | 0.0341 | 57.801 | 91058 | 0.0000 |
| weekdaySaturday:hour24 | 1.9362 | 0.0341 | 56.698 | 91058 | 0.0000 |
| weekdaySunday:hour6 | -2.1886 | 0.0350 | -62.483 | 91058 | 0.0000 |
| weekdaySunday:hour7 | -2.8615 | 0.0350 | -81.694 | 91058 | 0.0000 |
| weekdaySunday:hour8 | -1.1887 | 0.0350 | -33.937 | 91058 | 0.0000 |
| weekdaySunday:hour9 | 0.4213 | 0.0342 | 12.329 | 91058 | 0.0000 |
| weekdaySunday:hour10 | 0.8015 | 0.0342 | 23.457 | 91058 | 0.0000 |
| weekdaySunday:hour11 | 1.0983 | 0.0342 | 32.142 | 91058 | 0.0000 |
| weekdaySunday:hour12 | 1.0764 | 0.0342 | 31.501 | 91058 | 0.0000 |
| weekdaySunday:hour13 | 1.1364 | 0.0342 | 33.259 | 91058 | 0.0000 |
| weekdaySunday:hour14 | 1.0131 | 0.0342 | 29.651 | 91058 | 0.0000 |
| weekdaySunday:hour15 | 0.7738 | 0.0342 | 22.623 | 91058 | 0.0000 |
| weekdaySunday:hour16 | 0.7077 | 0.0342 | 20.691 | 91058 | 0.0000 |
| weekdaySunday:hour17 | 0.7232 | 0.0342 | 21.165 | 91058 | 0.0000 |
| weekdaySunday:hour18 | 0.9678 | 0.0342 | 28.324 | 91058 | 0.0000 |
| weekdaySunday:hour19 | 1.3228 | 0.0342 | 38.715 | 91058 | 0.0000 |
| weekdaySunday:hour20 | 1.4018 | 0.0342 | 40.985 | 91058 | 0.0000 |
| weekdaySunday:hour21 | 1.2722 | 0.0342 | 37.195 | 91058 | 0.0000 |
| weekdaySunday:hour22 | 1.3528 | 0.0342 | 39.553 | 91058 | 0.0000 |
| weekdaySunday:hour23 | 1.0835 | 0.0342 | 31.678 | 91058 | 0.0000 |
| weekdaySunday:hour24 | 0.8457 | 0.0344 | 24.591 | 91058 | 0.0000 |
| weekdayMonday:hour6 | 0.0434 | 0.0336 | 1.289 | 91058 | 0.1974 |
| weekdayMonday:hour7 | 0.0358 | 0.0336 | 1.063 | 91058 | 0.2879 |
| weekdayMonday:hour8 | 0.0074 | 0.0336 | 0.220 | 91058 | 0.8262 |
| weekdayMonday:hour9 | -0.0614 | 0.0336 | -1.828 | 91058 | 0.0675 |
| weekdayMonday:hour10 | -0.0493 | 0.0336 | -1.468 | 91058 | 0.1420 |
| weekdayMonday:hour11 | -0.0594 | 0.0336 | -1.770 | 91058 | 0.0767 |
| weekdayMonday:hour12 | -0.0882 | 0.0336 | -2.627 | 91058 | 0.0086 |
| weekdayMonday:hour13 | -0.1445 | 0.0335 | -4.311 | 91058 | 0.0000 |
| weekdayMonday:hour14 | -0.1204 | 0.0336 | -3.588 | 91058 | 0.0003 |
| weekdayMonday:hour15 | -0.0684 | 0.0335 | -2.041 | 91058 | 0.0412 |
| weekdayMonday:hour16 | 0.0719 | 0.0335 | 2.144 | 91058 | 0.0321 |
| weekdayMonday:hour17 | 0.1407 | 0.0336 | 4.192 | 91058 | 0.0000 |
| weekdayMonday:hour18 | -0.0587 | 0.0335 | -1.753 | 91058 | 0.0797 |
| weekdayMonday:hour19 | -0.2705 | 0.0336 | -8.062 | 91058 | 0.0000 |
| weekdayMonday:hour20 | -0.3797 | 0.0336 | -11.315 | 91058 | 0.0000 |
| weekdayMonday:hour21 | -0.4217 | 0.0336 | -12.566 | 91058 | 0.0000 |
| weekdayMonday:hour22 | -0.4171 | 0.0336 | -12.429 | 91058 | 0.0000 |
| weekdayMonday:hour23 | -0.6924 | 0.0336 | -20.633 | 91058 | 0.0000 |
| weekdayMonday:hour24 | -0.9700 | 0.0337 | -28.804 | 91058 | 0.0000 |
| weekdayTuesday:hour6 | 0.1540 | 0.0335 | 4.594 | 91058 | 0.0000 |
| weekdayTuesday:hour7 | 0.1670 | 0.0335 | 4.982 | 91058 | 0.0000 |
| weekdayTuesday:hour8 | 0.1151 | 0.0335 | 3.434 | 91058 | 0.0006 |
| weekdayTuesday:hour9 | 0.0157 | 0.0335 | 0.468 | 91058 | 0.6398 |
| weekdayTuesday:hour10 | -0.0226 | 0.0335 | -0.675 | 91058 | 0.4995 |
| weekdayTuesday:hour11 | -0.0677 | 0.0335 | -2.021 | 91058 | 0.0433 |
| weekdayTuesday:hour12 | -0.1068 | 0.0335 | -3.190 | 91058 | 0.0014 |
| weekdayTuesday:hour13 | -0.1380 | 0.0335 | -4.121 | 91058 | 0.0000 |
| weekdayTuesday:hour14 | -0.1307 | 0.0335 | -3.904 | 91058 | 0.0001 |
| weekdayTuesday:hour15 | -0.0841 | 0.0335 | -2.511 | 91058 | 0.0120 |
| weekdayTuesday:hour16 | 0.0344 | 0.0335 | 1.029 | 91058 | 0.3037 |
| weekdayTuesday:hour17 | 0.0966 | 0.0335 | 2.885 | 91058 | 0.0039 |
| weekdayTuesday:hour18 | -0.0509 | 0.0335 | -1.521 | 91058 | 0.1283 |
| weekdayTuesday:hour19 | -0.2729 | 0.0335 | -8.151 | 91058 | 0.0000 |
| weekdayTuesday:hour20 | -0.4232 | 0.0335 | -12.639 | 91058 | 0.0000 |
| weekdayTuesday:hour21 | -0.4636 | 0.0335 | -13.848 | 91058 | 0.0000 |
| weekdayTuesday:hour22 | -0.4439 | 0.0335 | -13.260 | 91058 | 0.0000 |
| weekdayTuesday:hour23 | -0.7372 | 0.0335 | -22.021 | 91058 | 0.0000 |
| weekdayTuesday:hour24 | -0.9771 | 0.0336 | -29.085 | 91058 | 0.0000 |
| weekdayWednesday:hour6 | 0.0823 | 0.0335 | 2.460 | 91058 | 0.0139 |
| weekdayWednesday:hour7 | 0.1155 | 0.0335 | 3.451 | 91058 | 0.0006 |
| weekdayWednesday:hour8 | 0.0520 | 0.0335 | 1.553 | 91058 | 0.1204 |
| weekdayWednesday:hour9 | -0.0103 | 0.0335 | -0.307 | 91058 | 0.7589 |
| weekdayWednesday:hour10 | -0.0628 | 0.0335 | -1.876 | 91058 | 0.0606 |
| weekdayWednesday:hour11 | -0.0533 | 0.0335 | -1.591 | 91058 | 0.1117 |
| weekdayWednesday:hour12 | -0.0443 | 0.0335 | -1.323 | 91058 | 0.1858 |
| weekdayWednesday:hour13 | -0.0815 | 0.0335 | -2.434 | 91058 | 0.0149 |
| weekdayWednesday:hour14 | -0.1428 | 0.0335 | -4.264 | 91058 | 0.0000 |
| weekdayWednesday:hour15 | -0.1088 | 0.0335 | -3.248 | 91058 | 0.0012 |
| weekdayWednesday:hour16 | 0.0294 | 0.0335 | 0.878 | 91058 | 0.3801 |
| weekdayWednesday:hour17 | 0.0804 | 0.0335 | 2.402 | 91058 | 0.0163 |
| weekdayWednesday:hour18 | -0.0712 | 0.0335 | -2.128 | 91058 | 0.0334 |
| weekdayWednesday:hour19 | -0.2550 | 0.0335 | -7.618 | 91058 | 0.0000 |
| weekdayWednesday:hour20 | -0.3503 | 0.0335 | -10.462 | 91058 | 0.0000 |
| weekdayWednesday:hour21 | -0.3953 | 0.0335 | -11.808 | 91058 | 0.0000 |
| weekdayWednesday:hour22 | -0.3408 | 0.0335 | -10.181 | 91058 | 0.0000 |
| weekdayWednesday:hour23 | -0.5701 | 0.0335 | -17.028 | 91058 | 0.0000 |
| weekdayWednesday:hour24 | -0.8065 | 0.0336 | -24.035 | 91058 | 0.0000 |
| weekdayThursday:hour6 | 0.0656 | 0.0334 | 1.964 | 91058 | 0.0495 |
| weekdayThursday:hour7 | 0.1068 | 0.0334 | 3.195 | 91058 | 0.0014 |
| weekdayThursday:hour8 | 0.0871 | 0.0334 | 2.609 | 91058 | 0.0091 |
| weekdayThursday:hour9 | 0.0523 | 0.0334 | 1.566 | 91058 | 0.1174 |
| weekdayThursday:hour10 | 0.0232 | 0.0334 | 0.695 | 91058 | 0.4871 |
| weekdayThursday:hour11 | -0.0177 | 0.0334 | -0.530 | 91058 | 0.5962 |
| weekdayThursday:hour12 | -0.0419 | 0.0334 | -1.254 | 91058 | 0.2100 |
| weekdayThursday:hour13 | -0.0839 | 0.0334 | -2.512 | 91058 | 0.0120 |
| weekdayThursday:hour14 | -0.0642 | 0.0334 | -1.923 | 91058 | 0.0544 |
| weekdayThursday:hour15 | -0.0270 | 0.0334 | -0.806 | 91058 | 0.4202 |
| weekdayThursday:hour16 | 0.0653 | 0.0334 | 1.952 | 91058 | 0.0509 |
| weekdayThursday:hour17 | 0.1271 | 0.0334 | 3.801 | 91058 | 0.0001 |
| weekdayThursday:hour18 | -0.0033 | 0.0334 | -0.099 | 91058 | 0.9213 |
| weekdayThursday:hour19 | -0.1888 | 0.0334 | -5.647 | 91058 | 0.0000 |
| weekdayThursday:hour20 | -0.2646 | 0.0334 | -7.913 | 91058 | 0.0000 |
| weekdayThursday:hour21 | -0.3018 | 0.0334 | -9.027 | 91058 | 0.0000 |
| weekdayThursday:hour22 | -0.2213 | 0.0334 | -6.619 | 91058 | 0.0000 |
| weekdayThursday:hour23 | -0.3640 | 0.0334 | -10.885 | 91058 | 0.0000 |
| weekdayThursday:hour24 | -0.5455 | 0.0335 | -16.296 | 91058 | 0.0000 |

# Appendix B: Bus use analysis: Robustness checks

Model 1 (Appendix A) is, however, just one of 2109 possible models to control for existing bus use patterns. These 2109 possible models are five models with only one control variable, one model with a five-way interaction between all control variables, 26 models that combine only main effects, 1023 models with 1 to 10 two-way interactions, 1023 models with 1 to 10 three-way interactions, and 31 models with 1 to 5 four-way interactions (models with higher-order interactions between a set of control variables always include the lower-order interactions between those control variables and the main effects of those control variables). To test whether the magnitude and statistical significance of the estimate of the interaction between condition and period — which tests whether the change in the number of rides from pre-intervention to post-intervention period is greater in the experimental than in the control condition — depends on the specification of the model controlling for existing bus use patterns, we carried out a specification curve analysis (Simonsohn, Simmons, & Nelson, 2020). In this analysis, we first fit a model with control variables to the data. The residuals of this model are then entered as dependent variable in a panel analysis with the main effects of, and interaction between, *condition* (*experimental* vs. *control*) and *period* (*post* if one day after the intervention or later, *pre* otherwise) as independent variables. This was done for a subset of all the possible models to control for existing bus use patterns, consisting of every model that has maximum three two-way interactions and every model that has maximum one three-way interaction (185 models in total). We did not test more complicated models due to computing power constraints.

The specification curve analysis shows that the estimate and the significance of the interaction term strongly depend on whether the interaction between *line* and *year* is included in the model. When this *line* $\times$ *year* interaction is included, the interaction between *condition* and *period* is essentially zero and non-significant (49 models, range of estimates = [-0.037, 0.004], minimum *p*-value = 0.081). This is probably because the *line* $\times$ *year* interaction largely coincides with the *condition* $\times$ *period* interaction (the *line* $\times$ *year* = 2016 terms are nested within the *condition* $\times$ *period* = ‘pre intervention’ terms and the *line* $\times$ *year* = 2018 terms are nested within the *condition* $\times$ *period* = ‘post intervention’ terms) and is fitted to the data first, therefore removing the effect that the *condition* $\times$ *period* interaction is testing. When the *line* $\times$ *year* interaction is not included, however, the interaction between *condition* and *period* is positive and strongly significant (136 models, range of estimates = [0.053, 0.058], maximum *p*-value = 0.022, median *p*-value = 0.0000007). Converting these estimates from the log-transformed back to the original scale gives us effect sizes similar to the one reported in the paper (range = [0.055, 0.059]).

Figure 3 shows an additional analysis. It shows the estimate and significance of the regression coefficient that represents the difference between experimental and control lines in the change in the number of rides from pre-intervention period to an *n*-day period right after the intervention, controlling for the same set of variables as specified in the main text. There are 147 possible choices for *n*. (We had data for 176 days after the intervention, but on 29 days, data was missing on one or more lines, rendering the data for those days unbalanced.) The graph shows the estimate for all choices of *n*. More formally, it shows the *condition* $\times$ *period* = 1 interaction term where *period* is 0 before the end of the intervention, 1 during the post-intervention period that starts on the first day after the intervention and ends after *n* days after the intervention, and 2 during the period that starts after *n* days after the intervention, and this for every possible choice of *n*. This figure shows that the estimate and significance of the regression term vary little with regards to *n*.


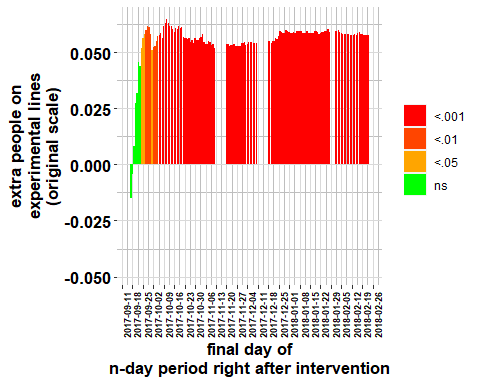


*Figure 3*. Estimate and significance of the difference between experimental and control lines in the change from pre-intervention period to the *n*-day period right after the intervention.
